# An appraisal of genetic testing for prostate cancer susceptibility

**DOI:** 10.1038/s41698-022-00282-8

**Published:** 2022-06-22

**Authors:** Amy Finch, Roderick Clark, Danny Vesprini, Justin Lorentz, Raymond H. Kim, Emily Thain, Neil Fleshner, Mohammad R. Akbari, Cezary Cybulski, Steven A. Narod

**Affiliations:** 1 Women’s College Research Institute, Women’s College Hospital, Toronto, Ontario, Canada; 2 Division of Urology, University of Toronto, Ontario, Canada; 3 Department of Radiation Oncology, Sunnybrook Health Sciences Center, University of Toronto, Ontario, Canada; 4 Familial Cancer Clinic, Princess Margaret Cancer Centre, University Health Network, Toronto, Ontario, Canada; 5 Department of Medicine, University of Toronto, Toronto, Ontario Canada; 6 Division of Urology, Departments of Surgery and Surgical Oncology, Princess Margaret Cancer Centre, University Health Network, University of Toronto, Toronto, Ontario Canada; 7 Dalla Lana School of Public Health, University of Toronto, Ontario, Canada; 8 Department of Genetics and Pathology, Pomeranian Medical University, Szczecin, Poland

**Keywords:** Cancer genetics, Cancer epidemiology

## Abstract

Most criteria for genetic testing for prostate cancer susceptibility require a prior diagnosis of prostate cancer, in particular cases with metastatic disease are selected. Advances in the field are expected to improve outcomes through tailored treatments for men with advanced prostate cancer with germline pathogenic variants, although these are not currently offered in the curative setting. A better understanding of the value of genetic testing for prostate cancer susceptibility in screening, for early detection and prevention is necessary. We review and summarize the literature describing germline pathogenic variants in genes associated with increased prostate cancer risk and aggressivity. Important questions include: what is our ability to screen for and prevent prostate cancer in a man with a germline pathogenic variant and how does knowledge of a germline pathogenic variant influence treatment of men with nonmetastatic disease, with hormone-resistant disease and with metastatic disease? The frequency of germline pathogenic variants in prostate cancer is well described, according to personal and family history of cancer and by stage and grade of disease. The role of these genes in aggressive prostate cancer is also discussed. It is timely to consider whether or not genetic testing should be offered to all men with prostate cancer. The goals of testing are to facilitate screening for early cancers in unaffected high-risk men and to prevent advanced disease in men with cancer.

## Introduction

Increasing numbers of men are encouraged to undergo germline genetic testing for prostate cancer. This is due in large part to better understanding of inherited prostate cancer risk and progression and an expansion of genetic services for cancer patients and for those at high risk of developing cancer.

Genetic testing for cancer has emerged over the last decade into panel-based testing whereby numerous genes are examined simultaneously for deleterious pathogenic/likely pathogenic variants or mutations. For example, most hereditary cancer panels include genes for breast, ovarian and colon cancer – and includes the five main genes for prostate cancer discussed here (*BRCA1*, *BRCA2*, *CHEK2*, *ATM*, *PALB2*). Most cancer susceptibility genes are associated with a predisposition to develop tumors at multiple sites. As a result, selecting individual genes for analysis is no longer a primary concern for the physician because patients are offered a ‘comprehensive cancer panel’ regardless of the indication.

Prostate cancer is the primary form of hereditary cancer which is specific to males. The main risk factors for prostate cancer are age, family history and race. Men of African descent are at greater risk for prostate cancer than Caucasian men, while men of Asian and Hispanic descent are at lower risk^1^. Men of African ancestry have an earlier age at diagnosis of prostate cancer, a higher incidence and increased prostate-specific mortality when compared with Caucasian men^1,2^. A genetic basis for the racial difference has not been established.

A man with a first-degree relative with prostate cancer has approximately twice the risk of a man who does not; this risk is higher if the relative is diagnosed under age 60^3–5^. This increased familial risk of prostate cancer is attributable in part to the additive effects of multiple low-risk germline variants (SNPs) and higher risk germline pathogenic variants in cancer susceptibility genes^6^. Polygenic risk scores (PRS) generated through combining a number of low-risk of prostate cancer susceptibility variants may provide discrimination in risk both for carriers of high-risk germline variants and for men in the general population^7–9^.

Our primary focus here is on moderate-to-high-risk hereditary cancer susceptibility genes. We discuss screening and prevention in men at high risk and the over-representation of these genes in aggressive prostate cancer. The individual genes are discussed below. Finally, we consider whether all men with prostate cancer should be offered genetic testing.

### Testing prostate cancer patients

There are several circumstances under which a man might benefit from genetic testing. For unaffected men, the information may help to specify his lifetime prostate cancer risk and aid in prevention and screening. For affected men, the information may be used to personalize cancer treatment. It is rare that an unaffected man will be the first to receive genetic testing in a family. For unaffected men, the most common scenario is that he is approached to initiate testing after a woman in his family is found to carry a *BRCA1* or *BRCA2* mutation (or a mutation in another gene). Many men describe being tested ‘for the sake of their daughters’ rather than for their own health^10^. For a prostate cancer patient, the opportunity to be tested will depend on his family history of cancer and his clinical presentation. To our knowledge, there is no guideline that suggests that all men with prostate cancer should be eligible for testing. Factors that determine eligibility are based on the probability of a mutation being detected (i.e. the prevalence) and include the presence of metastatic disease, high-grade disease, intraductal/cribriform histology, a family history of prostate cancer, a family history of breast or ovarian cancer, or a known mutation in the family^11^. If we consider the *utility* of testing as the primary goal of germline genetic testing we should address the following five questions:

1. What is our ability to prevent prostate cancer in men with a high risk of cancer?
2. What is the value of screening for prostate cancer among men at high risk?
3. Does a germline mutation influence prognosis?
4. How does the knowledge of a germline mutation influence treatment of men with nonmetastatic cancer?
5. How does the knowledge of a germline mutation influence treatment of men with metastatic cancer or with hormone-resistant disease?

For women, genetic testing for *BRCA1* and *BRCA2* mutations is now widely endorsed and accepted. Its value has been demonstrated through surgical prevention of breast cancer with prophylactic mastectomy, surgical prevention of ovarian cancer with bilateral salpingo-oophorectomy, the improved sensitivity of MRI over mammography screening, the benefit of MRI screening on breast cancer mortality and the benefit of specific chemotherapies, such as platinum-based neoadjuvant therapy for women with breast cancer and poly-ADP ribose polymerase (PARP) inhibitors (i.e. Olaparib) for treatment of women with ovarian cancer. It is now recommended that all women with epithelial ovarian cancer undergo genetic testing for susceptibility genes^11^. The clinical benefits of genetic testing for men for susceptibility genes is emerging but has not yet been established.

### What is the ability to offer prevention to unaffected men at high risk of prostate cancer?

Currently, there is no approved drug for the prevention of prostate cancer. Numerous randomized controlled trials have been performed on potential agents, including 5-alpha reductase inhibitors (which act to block the action of androgens in the prostate and include Dutasteride and Finasteride), medications, which manipulate the hormonal axis (e.g. Selective estrogen receptor modulator Toremifene), and non-steroidal anti-inflammatory drugs (e.g. Refocoxib). Several nutritional supplements have also been evaluated (e.g. selenium, vitamin E and soy products). Results of these trials have been disappointing, with one trial stopped because of cardiovascular toxicity (e.g Refocixib)^12^, and another showed an increase in prostate cancer risk (e.g. Vitamin E)^13^ and two trials, which showed a decline in overall prostate cancer risk in the treatment arm, but an increased risk of high-grade disease (PCPT and REDUCE trials)^14,15^. Given the lack of compelling evidence, none of these agents is currently recommended for prostate cancer prevention among individuals at average risk or high-risk. There are no trials underway specifically for chemoprevention in high-risk men, including carriers of high-risk pathogenic variants.

### What is the value of screening for prostate cancer among men at high risk?

The value of screening for prostate cancer in the general population is controversial. Three large randomized controlled trials of PSA screening and mortality produced mixed results. The European ERSPC study and the Gotenberg study found a 20–30% and 52% reduction in prostate cancer mortality respectively^16,17^, whereas the US PLCO trial showed no mortality difference between the treatment and control arms^18^. The US Preventative Services Task Force (USPSTF) recommends a discussion of the risks and benefits of PSA screening in men aged between 55 and 69 (and against screening for men over 70)^19^. The NCCN recommends that men with a germline mutation that increases the risk for prostate cancer consider annual PSA screening from age 40 (NCCN Guidelines V 2.2021 Prostate Cancer Early Detection)^20^. The NCCN also recommends annual screening in men with a family history and men of African descent from age 40, but notes that the evidence does not show a mortality benefit associated with earlier screening^2,20^. The American Cancer Society recommends starting a discussion about screening at the age of 40 for men at high risk, including those with more than one first-degree relative who had prostate cancer at an early age (a very small group)^21^.

The IMPACT study targets PSA screening specifically for male *BRCA1* and *BRCA2* mutation carriers (and controls) between ages 40 and 69. A biopsy is offered for those with a PSA > 3.0 ng/ml. A preliminary analysis demonstrated that men who carry a *BRCA2* mutation have a higher incidence of prostate cancer, a younger age at diagnosis and more clinically significant tumours than noncarrier controls. The IMPACT study group concluded that men who carry a *BRCA2* mutation should be offered annual PSA screening, but further follow-up is needed for *BRCA1* carriers^22^.

To some extent, the impact of PSA screening is limited by the sensitivity of the test and the possibility of diagnosing indolent disease. Alternative screening modalities include multiparametric magnetic resonance imaging (MRI). Segal et al. screened 188 *BRCA1 and BRCA2* carriers with no previous prostate biopsy with PSA and MRI^23^. Sixteen prostate cancers (8.5%) were diagnosed at the initial round of screening. MRI screening only missed one cancer (6%), while the PSA test alone missed five (31%). Further follow-up is needed to determine if there is an effect on mortality. The utility of screening in carriers will be determined when a large number of men who are identified to be at high risk are screened and followed.

### Does a germline mutation influence prognosis?

It is a central question whether or not the presence of a mutation predisposes men to more aggressive disease. There are several indicators of prostate cancer aggressivity that can be used in the evaluation. These include:

1. Gleason score (Grade group) at presentation.
2. The presence of metastatic disease at presentation.
3. Time to biochemical recurrence.
4. Treatment response.
5. Prostate-specific survival.
6. Time to death.
7. Mutation prevalence among men with metastatic disease *versus* localized disease.

It is important to ask if the prognosis of a man with an inherited pathogenic variant is influenced by the presence of the variant, independent of stage, grade and PSA level. It should also be noted that a pathogenic variant may not increase the risk of prostate cancer overall but may influence the probability of high-grade disease and prognosis to metastatic disease^24^. Based on limited studies to date, it appears that men with *BRCA2*, *ATM* and *PALB2* pathogenic variants face increased risks of progression and death that cannot be accounted for by traditional clinical parameters^25^. Further work is needed in this area.

### How does the knowledge of a germline mutation influence treatment of men with nonmetastatic cancer?

Prostate cancer accounts for 10% percent of all cancer deaths in men and its behavior varies from indolent to aggressive disease^26^. In a study of prostate cancer incidence and survival in the United States, localized, regional and distant-stage prostate cancer accounted for 77%, 11% and 5% of cases, respectively (cases with unknown stage accounted for 7%)^27^. Ten-year survival for localized prostate cancer was 100%, but was 96.1% and 78.1% for regional and unknown stage respectively. The five and 10-year survival for distant-stage prostate cancer was much lower at 30.7% and 18.5%, respectively. Current practice is to offer treatment according to recurrence risk, based on a combination of Grade group, PSA level and extent of disease. Germline mutation status is most often not considered in clinical practice guidelines at present but represents an opportunity to personalize treatment. For some genes (*BRCA2*, *PALB2* and *ATM*), germline mutations have been associated with aggressive disease and relatively poor outcomes^25^. Inherited variants in these DNA-repair genes are likely to play an increasingly important role in deciding the choice of treatment including the option of active surveillance^28–30^. Other genes have been less well studied or have been shown not to influence outcome.

Traditional treatments for localized prostate cancer include surgery (radical prostatectomy) and radiotherapy (external beam or brachytherapy). In general, surgery is favoured in younger men (<55 years old), radiotherapy is favoured in men over age 70 (in men between age 55 and 70 the decision is based on patient preference and tumour characteristics).

There are few studies that compare outcomes after surgery and radiotherapy for men with and without germline mutations. Castro et al. examined the tumor features and outcomes of 2,019 patients with prostate cancer, which included 18 *BRCA1* and 61 *BRCA2* carriers in a retrospective study^29^. They found that *BRCA2* mutation carriers were more likely to be diagnosed with high-risk disease (Gleason 8–10), have advanced clinical stage disease (T3/4), and/or have involvement of local lymph nodes or metastatic disease at diagnosis. Five-year cancer-specific survival was significantly worse for *BRCA* carriers than noncarriers (82% vs. 96%; *p* = 9 × 10^−8^). In a subsequent publication, Castro et al. compared survival among 67 *BRCA* carriers and 1235 noncarriers who received either radiotherapy or surgery for localized or locally advanced prostate cancer^31^. The groups were not directly comparable because there were a greater number of high-risk patients in the radiation therapy group than the radical prostatectomy group (69% vs. 34%). The 10- year metastases-free survival (MFS) was significantly lower for carriers than noncarriers in both the radical prostatectomy group (63% vs. 91%) and in the radiation therapy group (39% vs. 80%). Prostate-specific survival of carriers vs. noncarriers who underwent radical prostatectomy at 10 years was 79% vs 95% (log rank test *p* = 0.6). There was a significant difference in outcomes between carriers and noncarriers who had radiation therapy, with 10-year survival rates of 47% vs 81% respectively (log rank test *p* < 0.001). It is important that we compare the relative benefits of radical prostatectomy and radiation therapy directly among carriers with similar risk profiles in future studies.

### ATM and the risks of radiotherapy

A study of prostate cancer patients with heterozygous *ATM* mutations found an increased risk of late complications of external beam radiotherapy^32,33^. Others have demonstrated that there is potential for increased therapeutic efficacy of radiotherapy among *ATM* carriers, but care must be taken to minimize radiation dose to prevent toxicity and second malignancies^34^. At this time, the NCCN guidelines state that carrying a single *ATM* mutation should not lead to a recommendation to avoid radiation therapy^11^. There is little evidence regarding late toxicity and second malignancy of radiotherapy for carriers of other germline mutations.

### Active surveillance for low-risk disease

A historical overview of treatment outcomes of men with screen-detected, low-grade prostate cancer has led to the adoption of active surveillance for some low-risk prostate cancer patients. The choice of active surveillance relies on a combination of factors, including the PSA level, Grade group, clinical stage, and biopsy results (number of cores involved, percentage of involvement of dominant core, etc.). Active surveillance consists of PSA test every 3–6 months, digital rectal exam once per year, a confirmatory biopsy within 6–12 months, then serial biopsy a minimum of every 3–5 years thereafter with or without mpMRI^35^. In the event of a rising PSA or grade progression (e.g., Gleason 6 to ≥ 7) active treatment is offered. Between 36% to 73% of patients will transition to treatment over 10 years but the development of metastatic disease is rare^36,37^.

There is little data on the safety or efficacy of active surveillance for men diagnosed with a high-risk germline mutation. An important question is “Does the presence of a mutation predict for a worse outcome (in particular metastatic disease) after adjusting for the traditional clinical parameters (grade, PSA, screening history, etc.)? If so, active surveillance may not be appropriate for men with mutations. Carter et al. followed 1211 men with prostate cancer (from two cohorts) under active surveillance, including 26 with a mutation in *BRCA1*, *BRCA2* or *ATM*^28^. Eleven of 26 men with a mutation in one of the three genes experienced grade progression compared to 278 of 1185 noncarriers (adjusted hazard ratio = 2.0; (*p* = 0.05). This association was significant for *BRCA2* carriers (adjusted HR = 2.7; *p* = 0.01). The authors suggest that the mutation status should be considered in the decision to enter active surveillance.

### How does the knowledge of a germline mutation influence treatment of a man with metastatic cancer or with hormone-resistant disease?

After surgery or radiotherapy, patients may experience prostate cancer recurrence. Biochemical recurrence refers to a rising PSA level. Approximately 30–40% of patients will develop biochemical recurrence after radical treatment (e.g. surgery or radiotherapy)^38–41^. Biochemical recurrence is an early indicator of metastatic disease. The majority of men who experience a biochemical recurrence after surgery will develop metastatic prostate cancer within 10 years^42^. Common treatments for biochemical recurrence include radiotherapy and/or androgen deprivation (after surgery) and androgen deprivation therapy or surgery (after radiotherapy). Androgen deprivation therapy is generally not considered until a biochemical recurrence is documented. As previously discussed, individuals with a germline mutation in several genes (*BRCA2, ATM, PALB2*) are at increased risk for biochemical recurrence and prostate cancer-specific mortality. These men may potentially be candidates for earlier initiation of androgen deprivation therapy. Other options include cisplatin-based chemotherapy or early use of PARP inhibitors. While recent evidence has shown that these agents can be effective for women with breast cancer and a *BRCA* mutation^43^, and men with metastatic prostate cancer, more evidence is needed for high-risk men with localized prostate cancer.

Approximately 5–10% of men with prostate cancer present with metastatic disease^1^. The 5-year survival for men with metastatic prostate cancer is approximately 30%^44^, but recent advances in treatment are extending survival. The conventional treatment for metastatic prostate cancer is androgen axis inhibition, chemotherapy or radioligand treatments. Response to androgen deprivation therapy has led to the distinction between castrate-sensitive metastatic prostate cancer (responds to androgen blockage) and castrate resistant (continued progression of disease). Ten to twenty percent of patients with metastatic prostate cancer become castrate-resistant within 5-years^44^. Carriers of germline mutations are at greater risk of progressing from castrate-sensitive to castrate-resistant metastatic disease than noncarriers^45–47^.

PROREPAIR-B is an ongoing prospective study to evaluate the outcomes of patients with metastatic castrate-resistant prostate cancer with and without germline *ATM*/*BRCA1*/*BRCA2*/*PALB2* mutations^46^. They have demonstrated that patients with mutations in *BRCA2* have worse outcomes.

There is increasing interest in cisplatin-based chemotherapy and PARP inhibitors for metastatic prostate cancer^48^. Two PARP inhibitors (Olaparib and Rucaparib) are approved by the US Food and Drug Administration for men with metastatic castrate resistant prostate cancer (mCRPC) and a mutation in one of 15 homologous recombination repair genes (*BRCA1, BRCA2, ATM, BRIP1, BARD1, CDK12, CHEK1, CHEK2, FANCL, PALB2, PPP2R2A, RAD51B, RAD51C, RAD51D, and RAD54L)*^49,50^.

Men with a mutation in one of the MMR genes or with microsatellite instability-high/MMR-deficient tumours may benefit from anti-programmed cell death protein 1 (PD-1) antibody therapy^51^. Pembrolizumab was approved by the US Food and Drug Administration for the treatment of microsatellite instability-high (MSI-H) or mismatch repair–deficient (dMMR) solid tumors, including prostate cancer.

### Summary

There is currently no evidence for chemoprevention for prostate cancer.

-There is emerging evidence that screening unaffected men with a pathogenic mutations may have clinical utility and further research this area is required.

-There is emerging evidence that the presence of a mutation in *BRCA2*, *ATM* or *PALB2* puts the patient at increased risk for biochemical recurrence and metastatic disease.

-It is anticipated that knowing gene carrier status will allow more personalized discussions about prognosis, immediate management and management of metastatic and hormone resistant disease.

### Historical perspective

*BRCA2* was one of the first genes implicated in hereditary prostate cancer and is responsible for a significant proportion of hereditary prostate cancer cases around the world. Other genes implicated in the development of prostate cancer include those involved in Homologous Recombination DNA repair: *BRCA1* in 1994^52^, *CHEK2* in 2003^53,54^, *ATM* in 2004^55^, and *PALB2* in 2008^56^. The DNA mismatch repair genes that are associated with Lynch Syndrome (*MLH1, MSH2, MSH6 and PMS2*) have also been implicated^57^. Pathogenic variants in additional genes have been found at lower frequency including *HOXB13* and *RAD51D*^57^. These genes are associated with a range of cancer types. The exception is *HOXB13* which, to date has only been associated with prostate cancer.

### Frequency of germline mutations in men with prostate cancer

While germline mutations in genes that predisposes to prostate cancer have relatively low mutation prevalence individually, together they account for a significant proportion of prostate cancer cases. The prevalence depends on the population studied (e.g., family history, clinical presentation, means of selection) and the number of genes surveyed. In a cohort of 53,105 men with no diagnosis of cancer (general population from the Exome Aggregation Consortium), the frequency of DNA-repair gene mutations was 2.7%. Among 499 men with localized prostate cancer in the Cancer Genome Atlas Research Network the frequency of germline mutations was 4.6%. In a recent study of localized prostate cancer, 4% of men with European ancestry carried a DNA repair gene mutation compared 2.8% of unaffected controls^58^. Of 351 men with African ancestry and localized prostate cancer, 1.4% had a mutation. It is important to note that this study excluded men who later progressed to metastatic disease.

The prevalence rates are higher than this in men with metastatic disease and/or a family history of prostate cancer. In a study of 692 men with metastatic prostate cancer, 82 men (11.8%) were found to carry a mutation in one of 16 cancer susceptibility genes^56,59,60^ (See Table 1 for mutation frequency by gene). The frequencies of mutations in *ATM* and *BRCA1* and *BRCA2* were compared in 313 men who died of prostate cancer and 486 men with localized prostate cancer^61^. The mutation rate was much higher among those with lethal prostate cancer (6.1% vs 1.4%, *p* = 0.0007). For those who died below age 60, 10% carried a mutation. Of those who died within five years of diagnosis 12.3% carried a mutation.

**Table 1:** Germline Mutation frequency in Men with Prostate Cancer and Population Controls.

	Exome Aggregation Cohort General Population ī = 53,105 (%)	Lee et al.^58^ Localized Prostate Cancer *ī = 1174 European population (%)	TGCA cohort Localized Prostate Cancer ī = 499 (%)	Leongamornlert et al.^59^ Familial Prostate Cancer ī = 191 (%)	Prichard et al.^56^ Metastatic Prostate Cancer ī = 692 (%)	Castro et al. Prorepair-B^46^ Metastatic CR Prostate Cancer ī = 419 (%)	Nicolosi et al.^62^ Invitae Prostate Cancer** ī= 3607 (%)
All Genes: % +ve	2.7%	4.0%	4.6%	7.3%	11.8%	16.2%	17.2%
BRCA1	0.22	0.77	0.60	0.52	0.87	1.0	1.25
BRCA2	0.29	1.0	0.20	2.10	5.35	6.2	4.74
ATM	0.25	0.51	1.0	1.04	1.59	1.9	2.03
PALB2	0.12	0.17	0.40	0.52	0.43	–	0.56
CHEK2	0.61	0.34	0.40	1.04	1.87	0.24	2.88
MLH1	0.02	0.0	0	–	0	–	0.06
MSH2	0.04	0.17	0.20	–	0.14	0.24	0.69
PMS2	0.11	0.09	0.20	0.52	0.29	–	0.54
MSH6	0.08	0.09	0.20	–	0.14	–	0.45
HOXB13	–	–	–	–	–	–	1.12
RAD51D	0.08	0.0	0.20	–	0.43	–	0.15

^*^Men who subsequently developed metastatic disease were excluded

^**^Referral-based genetic testing for personal history of prostate cancer and includes men with additional risk factors for hereditary cancer.

In the UK Genetic Prostate Cancer Study (UKGPCS), 191 men with prostate cancer with two or more relatives with prostate cancer were tested for mutations in 22 tumour suppressor genes. Fourteen men (7.3%) were found to carry a mutation in one of eight genes^59^. Nicolosi et al. reported on a large series of 3607 men with prostate cancer with varying family history and ethnicity tested by Invitae^62^. The overall mutation detection rate reported was considerably higher than for previous studies (17.2%), but these patients were selected and referred for testing. Many had a family history of cancer. The highest mutation frequencies were found in men with prostate cancer and a family history of ovarian cancer (22.8%), a family history of pancreatic cancer (19.4%) and Ashkenazi Jewish ancestry (22.2%).

In the next section we consider prostate cancer susceptibility genes individually.

### BRCA1

*BRCA1* predisposes women to breast, ovarian, fallopian tube and peritoneal cancer^52,63^. An early study of prostate cancer in *BRCA1* carriers found an of elevated risk for prostate cancer among those under age 65 with a relative risk of 1.82 (95% CI: 1.01–3.29, *p* = 0.05)^64^. This was confirmed in a second series of 913 men with clinically evident prostate cancer (RR = 3.75 (95% CI: 1.02–9.6)) translating to approximately 8.6% risk to age 65^65^. A recent prospective study of prostate cancer risk found men who carry a *BRCA1* mutation have an absolute risk for prostate cancer of 21% to age 75 and 29% to age 85^66^. Among men with metastatic prostate cancer, approximately 1% carry a *BRCA1* mutation^46,56^. It is not clear that *BRCA1* predisposes to a more aggressive form of prostate cancer.

### BRCA2

*BRCA2* is the best-established prostate cancer susceptibility gene. *BRCA2* also predisposes women to breast and ovarian cancer (including fallopian tube and peritoneal cancer)^67^. The risks for pancreatic cancer, melanoma, and for men, prostate and breast cancer, are also higher than general population risk^64,68^. Kote-Jarai et al. estimated that *BRCA2* confers an approximately 8.6 fold increase in prostate cancer risk by age 65, with a 15% absolute risk by age 65^69^. In the prospective study by Nyberg et al., *BRCA2* carriers had an absolute risk of prostate cancer of 27% to age 75 and the risk estimate reached 60% by age 85. To some extent these risk estimates depend on the intensity of surveillance.

Among men with *BRCA* mutations, the family history impacts upon risk. For *BRCA1* carriers, risk for prostate cancer was higher for those with a positive family history of prostate cancer (SIR = 3.2) than for those without (SIR = 2.3). For *BRCA2* carriers the SIR was 7.3 for those a positive family history and was 3.9 for those without^66^.

The *BRCA2* mutation frequency is similar for men with early-onset prostate cancer (<age 55) (2.3%) and for men with familial prostate cancer (3 or more cases of prostate cancer) (2.1%)^59^. The frequency of *BRCA2* mutations was 4.5% in a large series of men with prostate cancer referred for testing with varying family histories, ethnicities and stages of disease^62^.

Multiple studies have found poor survival for *BRCA2* carriers compared with noncarriers^28,29,61,70–72^ *BRCA2* carriers have a stronger association with Gleason score ≥ 7 cancer than with Gleason score ≤ 6, cancer and a higher risk of death from prostate cancer^66^. Castro et al. examined 2019 men with prostate cancer (18 *BRCA1* carriers, 61 *BRCA2* carriers and 1940 noncarriers) with the aims of determining the independent prognostic value of *BRCA1* and *BRCA2* mutations on prostate cancer characteristics and survival^29^. They found that carriers were more likely to have poorly differentiated cancer (Gleason score ≥ 8) (35% vs 15%; *p* = 0.00003), advanced stage (T3-T4) (37% vs 28%; *p* = 0.003) and metastatic spread (M1:18% vs. 9%; *p* = 0.005). Median cause-specific overall survival was shorter in carriers than noncarriers (8.6 vs. 15.7 years; *p* = 7 × 10^−8^). A trend toward better median cause-specific survival was observed in BRCA1 carriers compared with BRCA2 but it was not significant (10.5 and 8.6 years, *p* = 0.37). The 5-year metastases-free survival was also lower in carriers than in noncarriers (77% vs. 93%; *p* = 0.0001). In the Prorepair-B study of men with Spanish ancestry and metastatic castrate-resistant prostate cancer 3.3% carried a *BRCA2* mutation, slightly lower than the 5.4% among the 692 men with metastatic prostate cancer in the study by Pritchard et al^46,56^. *BRCA2* accounted for 44% of all pathogenic variants detected among the men with metastatic cancer in the Pritchard study.

### ATM

The *ATM* gene (Ataxia Telangiectasia Mutated) is required for cell response to DNA damage and for genome stability. Women who carry an *ATM* mutation have a 20–30% lifetime risk to develop breast cancer, and carriers also have a possible but as yet undefined risk for other cancers, such as prostate, pancreatic and colon cancer^73–75^. The frequency of *ATM* mutations ranges from approximately 0.5% in the general population (Exome Aggregation Consortium), to 1% in men with localized prostate cancer to 1.6% in men with metastatic prostate cancer^56,60^. In addition, *ATM* mutations have been associated with aggressive disease, and with metastatic disease^28,56^. Darst et al. performed a case-control study of aggressive vs. nonaggressive prostate cancers in men of European ancestry^76^. They found *ATM* was associated with aggressive disease, with 1.6% of aggressive and 0.8% of nonaggressive cases carrying a pathogenic or likely pathogenic variant (OR = 1.88, 95% CI : 1.10–3.22, *p* = 0.02). In Poland, 50% of cancers diagnosed among *ATM* carriers were Gleason score 8–10, compared to 22.7% in noncarriers (*p* = 0.03)^77^.

### CHEK2

*CHEK2*, also called cell cycle checkpoint kinase 2 is a cell cycle check point regulator. The variant c.1100delC accounts for the majority of pathogenic variants in this gene^78^. *CHEK2* mutations confer an increased risk for breast (in both males and females), thyroid, colon, and prostate cancer^78–82^. Hale et al. pooled five studies and examined prostate cancer risk associated with the 1100delC variant. They found the odds ratios were 1.98 (95% CI: 1.23–3.18) for unselected cases and 3.39 (95% CI: 1.78–6.47) for familial cases^83^. Cybulski et al. examined prostate cancer risk associated with the truncating variant 1100delC in addition to two other truncating variants (IVS2 + 1 G > A (c.444 + 1 G.A), del5395) and one missense variant (p.I157T). The p.I157T missense variant was associated with an odds ratio of 1.8 (95% CI: 1.5–2.2), *p* < 0.0001, as compared with the truncating variants combined (OR = 2.1 (95% CI: 1.4–3.0), *p* = 0.0001. For the 1100delC variant alone the odds ratio was 3.2 (95% CI: 1.4–7.5), *p* = 0.009^84^.

*CHEK2* variants are found in fewer than one percent of the general population (0.61% in the Exome Aggregation Consortium) and in two percent of men with metastatic prostate cancer, giving a relative risk for metastatic prostate cancer of 3.1 (95% CI: 1.5–5.6), *p* = 0.0002^56^. *CHEK2* mutations were among the most commonly found variants in men with metastatic cancer (12% of 84 variants detected among 692 men with metastatic disease)^56^.

### PALB2

*PALB2* is a known breast cancer susceptibility gene with risk of breast cancer estimated to be approximately 14% to age 50 and 53% to age 80^85^. It is also associated with a moderate risk of ovarian cancer (approximately 5% to age 80)^86^. Risks may also be increased for pancreatic cancer (2–3%) and male breast cancer (1%). An association with gastric cancer has also been reported^87,88^.

Studies of families with mutations in *PALB2* have not demonstrated an increased risk for prostate cancer, but *PALB2* variants have been associated with aggressive prostate cancer and with metastatic prostate cancer^24,25,86^. Pritchard et al. found an increased frequency of *PALB2* mutations in the series of 692 men with metastatic prostate cancer when compared with a large population of people unaffected with cancer (RR 3.5, 95% CI: 0.7–10.3, *p* = 0.05)^56^. Wokolorczyk et al tested 5472 unselected Polish men with prostate cancer and 8016 Polish controls for two founder *PALB2* variants. They found that *PALB2* carriers were not more common in prostate cancer patients overall, but were common in those diagnosed with aggressive prostate cancers of high grade (Gleason score 8–10) than noncarriers (64.3% vs. 18.1%, *p* < 0.0001). The five-year survival for *PALB2* carriers was 42% compared with 72% for noncarriers (*p* = 0.006)^24^.

### MMR genes

The mismatch repair (MMR) genes (*MLH1*, *MSH2*, *MSH6*, *PMS2*) play a key role in genomic stability and are associated with significantly increased risks varying by gene for colon cancer (15–46%), endometrial cancers (43 to 57%) and ovarian cancer (10–17%)^89–91^. Mutations in MMR genes are associated with additional cancers such as urothelial, gastric, small bowel, pancreatic, hepatobiliary, brain, and sebaceous carcinomas^89,92^. Germline mutations in these genes are collectively known to cause Lynch syndrome.

In a study of Lynch Syndrome families, the cumulative lifetime risk (to age 80) of prostate cancer was estimated to be 30% in MMR mutation carriers^57,93^. *MSH2* was the most strongly associated with prostate cancer of the MMR genes, with a risk of 23.8% risk to age 75 in the Prospective Lynch Syndrome Database^94,95^. Similarly, Barrow et al found a 10-fold increased risk of prostate cancer associated with *MSH2* in study of male Lynch syndrome mutation carriers and their first-degree male relatives who were part of the Manchester Regional Lynch Syndrome database (RR 10.41; 95% CI: 2.80–26.65)^89^. In the series of 3607 men with prostate cancer, 2% carried a mutation in one of four DNA mismatch repair genes (*MLH1*, *MSH2*, *MSH6* and *PMS2*). *MSH2* accounted for the greatest proportion of these mutations followed by *PMS2* and *MSH6* with only 2 of 58 pathogenic variants in these 4 genes identified in *MLH1*^62^. In studies to date, the age at onset and aggressiveness associated with MMR-related prostate cancers do not appear to be different than observed in sporadic cases^96^.

### RAD51D

*RAD51D* is involved in homologous recombination repair and is primarily associated with hereditary ovarian cancer, and to a lesser extent, breast cancer^78,79^. Germline *RAD51D* mutations are relatively rare in men with prostate cancer, (3/692 = 0.43%). Although the mutation frequency is low, the relative risk of metastatic disease was 5.7 (95% CI: 1.2–16.7, *p* = 0.02)^56^. Further studies are needed to better define the role of *RAD51D* in the development of aggressive and metastatic prostate cancer.

### HOXB13

*HOXB13* is a homeobox transcription factor gene and a single rare variant G84E confers a significantly increased risk for prostate cancer^97,98^ The odds ratios for development of associated with this variant range from 2.9 (95% CI: 1.9–4.6) in the UK Genetic Prostate Cancer Study (UKGPCS) to 8.8 (95% CI: 4.9–15.7) among men with a family history in a Finnish study by Laitinen et al.^99,100^. Nyberg et al estimated the absolute risk of prostate cancer in a large UK-based kin-cohort study to be 60% to age 85 among men with the G84E variant with no family history and 98% for men with the variant and two relatives with prostate cancer at young ages, compared to 15% for noncarriers^101^. In Poland the G84E allele was associated with a five-fold increase in the risk of unselected prostate cancer^102^.

In the Finnish study, 8.4% (16 of 190) men with prostate cancer and a positive family history were positive for the *HOXB13* variant, higher than observed in other cohorts of familial prostate cancer but similar to that in Sweden, suggesting a founder effect for the variant^100^. The International Consortium for Prostate Cancer Genetics (ICPCG) genotyped a large sample of prostate cancer families and found the variant was present in approximately 5% of prostate cancer families of European descent^99^. The *HOXB13* variant has been found to be associated with younger age at diagnosis and higher PSA values at diagnosis^100,103,104^. There is little data to date on the presence of *HOXB13* mutations in non-European populations.

### Prostate cancer: who to test

Current guidelines for germline genetic testing tend to be complex and focus on men with prostate cancer. The NCCN Guidelines (V 2.2021 for Prostate Cancer and the NCCN Guidelines V2.2021 for High-Risk Assessment for Breast, Ovarian and Pancreatic Cancer) recommend germline genetic testing for patients with prostate cancer who satisfy additional criteria such as Ashkenazi Jewish ancestry, family history of cancer and/or based on characteristics of the prostate cancer, such as grade, stage, histology and the presence of metastatic disease. A man with a likelihood 2.5–5% of carrying a pathogenic *BRCA1/2* variant (in prior probability models such as Tyrer Cuzick, BRCAPro, CanRisk) is also eligible for testing^11^. The likelihood of detecting a germline pathogenic variant in men with prostate cancer are presented by gene and by study in Table 1. All genes listed in Table 1 are considered to be actionable hereditary cancer predisposition genes, but not all genes pose similar risk for prostate cancer or for more aggressive disease. The genes which are believed to have the greatest impact in terms of risk are highlighted in Table 2 (*BRCA1, BRCA2, ATM* and *PALB2*). *HOXB13* is not included in many of these studies but is associated with prostate cancer in European populations.

**Table 2:** The prevalence of mutations by various at risk groups.

Group	Prevalence of LP/P variant	Prevalence of LP/P variant in: BRCA1/BRCA2, ATM & PALB2
Men diagnosed with localized prostate cancer	4.0%^58^, 4.6%^60^	2.5%^58^, 2.2%^60^
Men diagnosed with metastatic prostate cancer	11.8%^56^	8.2%^56^
Affected men with 2 relatives with prostate cancer	7.3%^59^	4.2%^59^
Men with first degree relative with epithelial ovarian cancer	10% (for BRCA only^109^)	10% (for BRCA only^109^)

The prevalence of mutations according to risk group is also presented in Table 2. The prevalence is highest for men with a female relative with ovarian cancer and for men with metastatic prostate cancer. The majority of mutations are in *BRCA1* and *BRCA2*, although the number of genes tested varies from study to study.

The 2019 Philadelphia Prostate Cancer Consensus Conference on the implementation of Germline Genetic Testing for Prostate Cancer identified challenges in the implementation of testing in three main areas: determining which genes should be included in panels, variability in guidelines regarding who should have genetic testing and the availability of genetic services^30^.

Population testing of all men will identify the greatest number of unaffected men at risk for prostate cancer and allow for targeted screening and possibly prevention; however, this is not currently feasible. A focused approach is required. Genetic testing of patients allows an opportunity to identify those who are more likely to progress to advanced or metastatic disease and provides and opportunity for intervention. Testing for underlying hereditary predisposition has become standard of care for patients with other cancers, such as colon cancer and epithelial ovarian cancer^11,105^. Goals for genetic testing for prostate cancer should include prevention of progression of the disease in men at highest risk and improvement in treatment of hereditary prostate cancer. Currently testing is not recommended for all men with prostate cancer, rather is based on the probability that a mutation is likely to be found. In the event that personalised mutation-specific treatments for prostate cancer are developed, there will be an impetus to expand testing to more prostate cancer patients. This will also allow the identification of other family members at risk for a range of cancers (cascade testing).

#### ***Models of genetic testing***

Prostate cancer is very common in Europe and North America and if genetic testing is to be expanded to more men with prostate cancer, it is important to consider how to do it effectively and efficiently. The current model of genetic services includes pre- and post-testing genetic counselling and is time consuming and expensive. There are too few genetic counsellors to expand this model to testing all prostate cancer patients and their specialized skills and training are most needed to support patients who receive a positive genetic test result. To expand genetic testing, other options should be considered. Many models are being explored and implemented including direct-to-consumer genetic testing, sponsored genetic testing, and physician-mediated genetic testing (mainstream testing). Each model has unique challenges. McCuaig et al. provide an informative summary of this in regard to testing all women with epithelial ovarian cancer; these considerations also apply to men with prostate cancer^106^.

**TheScreenProject** is an example of a guided direct-to-consumer genetic testing model for *BRCA1* and *BRCA2*, with the patient given the option of expanded panel testing^107^. All men and women over age 18 are eligible to self-refer to the study including those with and without cancer. The test is available to all men with prostate cancer, regardless of clinical presentation or family history. They register online, are invited to watch a pre-test counselling video, provide informed consent online, pay a fee for testing and are sent a saliva kit to submit their DNA for testing. A genetic counsellor is available throughout the process to all participants. Those participants who receive a positive test result are contacted by the study genetic counsellor and referred to a local clinic for counselling and follow-up. Where available, men with a positive result are referred to a high-risk clinic that provides both clinical screening and opportunities for further research studies^108^. In the first phase of the study the overall satisfaction with the process was very high. Only 5% of participants with a negative result requested genetic counselling. The principal difference between **TheScreenProject** and other Canadian testing programs is that the patient is required to pay 250 USD to be tested whereas provincial programs are without cost to the patient (if eligible).

## Conclusions

The genetic basis of prostate cancer has been well established, the most relevant genes identified and we are now in the position to translate this information into better cancer care. Challenges include identifying more men who carry a cancer predisposing mutation, in particular prior to a cancer diagnosis or at the time of early diagnosis. Genetic testing should be done according to the presentation of prostate cancer and the family history of prostate and ovarian cancer or where there is a known mutation in the family. The majority of the genes involved in homologous recombination repair which predispose women to breast cancer are also associated with high Gleason grade prostate cancer and with a propensity to metastasize. It is probably unwise to consider these men as candidates for active surveillance.

There are several priority areas for further research:

To identify means to prevent hereditary prostate cancer.

To estimate the mortality rates for men with localised prostate cancer and a susceptibility gene.

To determine the impact of screening with PSA and MRI on mortality.

To compare the outcomes of radical prostatectomy and radiotherapy in men with inherited susceptibility mutations.

To develop personalised treatment options. There is evidence that specific treatment (i.e. olaparib) may be warranted for *BRCA2* carriers but this is not yet the case for men with mutations in other genes.

It is important that collaborative observational studies are conducted which follow men with mutations in order to evaluate various prevention and treatment options. Translational research is needed so that the benefits of genetic testing that have been realized for women are realized for men. The goal should be to expand our current focus from the treatment of men with metastatic disease towards prevention, screening and the treatment of early-stage disease.
